# Commissioning and end‐to‐end validation of a combined surface‐guided and triggered kV imaging workflow for breath‐hold SBRT on a Varian TrueBeam

**DOI:** 10.1002/acm2.70812

**Published:** 2026-09-18

**Authors:** Offormata E. Osunkwor, Alois Ndlovu, Roland F. Teboh

**Affiliations:** ^1^ John Theurer Cancer Center Hackensack University Medical Center Hackensack New Jersey USA

**Keywords:** Commissioning, DIBH, Motion management, SBRT, SGRT

## Abstract

**Background:**

Respiratory motion introduces significant geometric uncertainty in Stereotactic Body Radiotherapy (SBRT) for thoracic and abdominal tumors. Deep Inspiration Breath‐Hold (DIBH) mitigates this, but the surface signal alone may not reflect internal target position, supporting the need for real‐time internal‐anatomy verification during delivery.

**Purpose:**

To commission a Surface‐Guided Radiation Therapy (SGRT) system and validate an integrated SGRT + Image‐Guided Radiation Therapy (IGRT) + triggered kV imaging (SITI) workflow for DIBH SBRT.

**Materials & Methods:**

The LAP LUNA 3D SGRT system was commissioned per AAPM TG‐302 using phantom‐based assessment of static and dynamic localization accuracy, reproducibility, and latency. An end‐to‐end test using a dynamic phantom validated the SITI workflow. Point dose was measured with an ion chamber under four scenarios: SGRT‐only delivery, full SITI delivery, and SITI with induced 1 and 2 mm uncorrected 3D shifts.

**Results:**

Static localization accuracy was better than 0.5 mm / 0.3°, with reproducibility within 0.2 mm / 0.1°. Dynamic testing confirmed sub‐millimeter spatial accuracy and a 31.6 ms latency, well below the AAPM TG‐302 100 ms tolerance. The 1 and 2 mm uncorrected shifts produced point‐dose reductions of 0.9% and 2.2% relative to the SITI reference, consistent with the ∼1%/mm local dose gradient. Both shifts were clearly visualized on triggered kV images, confirming detection of sub‐tolerance residual displacements undetected by surface guidance alone.

**Conclusions:**

The LAP LUNA 3D system meets the technical requirements for SBRT. The SITI workflow is technically feasible and provides real‐time visualization of internal target position during delivery, offering a robust motion‐management strategy for DIBH SBRT of mobile thoracic and abdominal targets.

## 1 INTRODUCTION

Respiratory motion remains a primary source of geometric uncertainty in the radiation treatment of thoracic and abdominal tumors. This uncertainty is particularly critical in Stereotactic Body Radiotherapy (SBRT), where high dose gradients mean that a target miss can lead to significant underdosing of the tumor and simultaneous overdosing of adjacent healthy tissues.^1^, ^2^, ^3^, ^4^ Consequently, robust motion management strategies are essential for the safe and effective delivery of SBRT to moving targets.

Several motion management strategies have been developed to compensate for respiratory‐induced tumor motion. The most common is the Internal Target Volume (ITV) approach, where the full extent of tumor motion is encompassed in the treatment volume. While straightforward to implement, this method often leads to irradiation of a substantially larger volume of normal tissue, particularly in the abdomen where motion amplitudes can be significant.^5^, ^6^ Respiratory gating and tumor tracking reduce PTV margin but increase treatment time and risk geometric miss if the tumor–surrogate correlation drifts.^7^, ^8^ Abdominal compression can reduce motion amplitude but is often limited by patient tolerance. ^9^, ^10^

Among these options, breath‐hold (BH) techniques offer a compelling balance, reducing the PTV margin compared to an ITV approach without the reduced duty cycle inherent to gating. In thoracic cases, deep inspiration breath‐hold (DIBH) also has the dosimetric benefit of reducing the relative volume and density of irradiated lungs. However, recent studies have demonstrated that significant residual tumor motion can persist during BH, even when the external surface appears stationary.^4^, ^11^, ^12^, ^13^, ^14^ This highlights the limitation of using the patient's surface as a surrogate for internal tumor position and raises concerns about intrafractional BH variability, especially during multi‐arc SBRT deliveries that require numerous successive BHs.

This challenge necessitates real‐time, internal anatomy verification during beam delivery to ensure treatment fidelity. Zeng et al.^15^ directly demonstrated this need in 14 gastrointestinal DIBH VMAT patients, reporting residual internal motion of 3–21 mm despite a ± 3 mm/ ± 3° external surface tolerance‐with instantaneous internal displacement exceeding 5 mm in 19% of triggered images, and explicitly recommended real‐time radiographic verification as a safety prerequisite for SGRT‐based DIBH where the surface‐to‐internal correlation is limited. One commercial response is the ExacTrac Dynamic (EXTD) system (Brainlab AG, Munich, Germany), which integrates optical/thermal surface imaging with stereotactic kV X‐ray imaging in a single dedicated platform.^16^ EXTD, however, is a vendor‐specific standalone solution that may require dedicated hardware that may not be feasible for some clinics operating a Varian TrueBeam, with triggered‐imaging capabilities.

Building on these rationales, the present study proposes and evaluates a workflow that achieves combined surface and internal‐anatomy verification using existing Varian TrueBeam infrastructure rather than a dedicated hardware. The proposed Surface‐Guided Radiation Therapy (SGRT) + Image‐Guided Radiation Therapy (IGRT) + Triggered Imaging (SITI) workflow integrates the LAP LUNA 3D SGRT system (LAP GmbH, Germany), with the TrueBeam's native CBCT and triggered kV imaging (Varian, Palo Alto, CA), in which triggered imaging provides periodic internal‐anatomy “snapshots” during beam delivery based on gantry angle, time, or monitor units delivered.14

As recommended by AAPM TG‐302 17 and ESTRO‐ACROP guidelines,18 comprehensive commissioning is required before the clinical implementation of any SGRT system.^17^, ^18^ Published commissioning data for the LUNA 3D system remain limited; while sub‐millimeter tracking accuracy has been demonstrated,^19^ a comprehensive characterization of its localization and temporal performance was a prerequisite for its inclusion in the SITI workflow.

In our clinic, triggered kV imaging is routinely used for intrafraction motion verification during SBRT of thoracic and abdominal tumors. The approach relies on high‐density internal markers (e.g. fiducial) near or within the tumor as high‐contrast internal surrogates, with kV projection images acquired at pre‐defined intervals during beam delivery and the planned internal marker contour superimposed on the live image for verification of target localization.

By integrating DIBH with this triggered‐imaging practice, the proposed workflow combines the smaller PTV margins of DIBH planning with real‐time internal verification during delivery. To our knowledge, this is the first reported commissioning of the LAP LUNA 3D SGRT system for SBRT, and the first end‐to‐end (E2E) validation of an SGRT + IGRT + triggered‐imaging workflow built around this system on the Varian TrueBeam without dedicated proprietary hardware.

## 2 MATERIALS AND METHODS

### 2.1 SGRT commissioning and equipment

A set of commissioning tests was designed to evaluate the LUNA 3D system's functionality for SBRT use, guided by AAPM TG‐302 recommendations. The main tests reported here are static and dynamic localization accuracy and reproducibility, and a comprehensive end‐to‐end test evaluating the operational feasibility and dosimetric consistency of the integrated SITI workflow.

**FIGURE 1:**
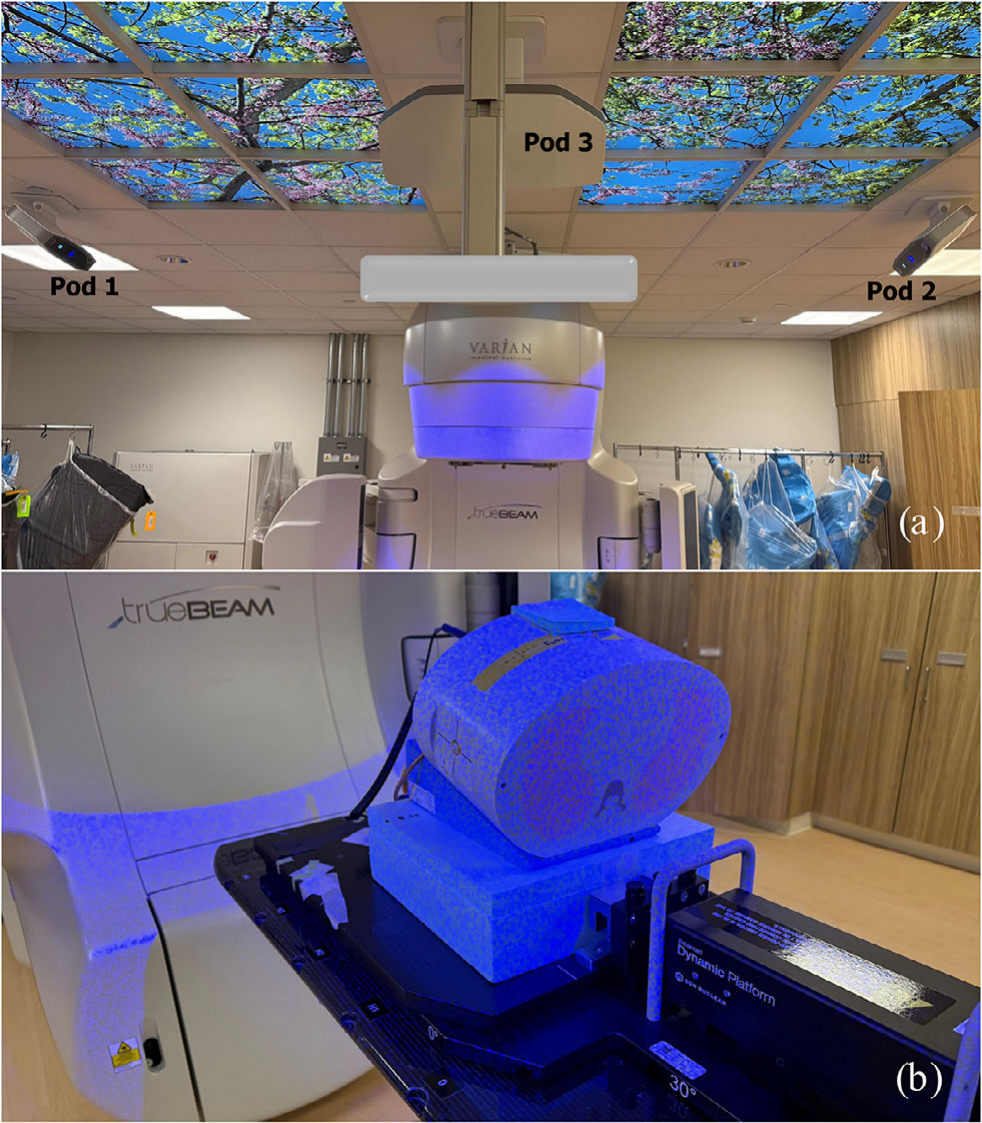
(a) The LUNA 3D SGRT system showing the three camera pods. (b) Phantom setup for the dynamic delivery test at CT simulation; the wedge simulates vertical motion (platform is longitudinal only).

The treatment delivery system was a Varian TrueBeam v2.7 (Varian Medical Systems, Palo Alto, CA) with the advanced imaging package, equipped with CBCT and triggered kV imaging. The LUNA 3D SGRT system consists of three camera pods, each containing a projector that produces a pseudo‐random speckled light pattern based on the principle of photogrammetry and two camera units (Figure 1a). The use of multiple pods and cameras maximizes the field of view, reducing self‐occlusion and gantry obstruction so that a given region of interest (ROI) remains visible under various treatment conditions. The current version of the system is not interfaced with the TrueBeam; therefore, automatic beam hold is not available, and the beam must be held manually when SGRT tolerances are exceeded.

### 2.2 Static localization reproducibility

The CIRS E2E SBRT phantom, Model 036A (Sun Nuclear, Melbourne, FL; Figure 1b), was aligned to the room lasers at couch angle zero, and the SGRT system captured a live reference surface via the camera pods. Known translational and rotational shifts from isocenter were then applied to the couch and the SGRT real‐time deltas (RTD) recorded for each shift.

### 2.3 SGRT dynamic spatial localization accuracy and latency

For DIBH treatment using LAP LUNA 3D SGRT, the free‐breathing (FB) and BH reference body contours can be automatically used to set the target threshold for patient feedback, ensuring the same BH level established during CT simulation. High dynamic accuracy, both spatial and temporal, is therefore critical to achieve reproducible BHs and accurate target localization.

Both the dynamic spatial accuracy and the system latency were evaluated in a single experiment. The CIRS E2E SBRT phantom was placed on the Enhanced Dynamic Platform, Model 008PLX (Sun Nuclear, Melbourne, FL), which was programmed with a sinusoidal waveform of 10 mm amplitude and 5 s period in the longitudinal direction. The longitudinal axis was selected because it represents the predominant direction of respiratory‐induced target motion for lung and abdominal tumors — the principal clinical indication for the workflow evaluated in this study — and is also the axis along which triggered imaging provides continuous in‐plane resolvability at every gantry angle. Before activating the motion, a live reference surface of the phantom was captured using SGRT and a treatment ROI was drawn.

Dynamic spatial accuracy was determined by comparing the amplitude of the motion trace reported by the SGRT system with the known programmed amplitude. The dynamic spatial localization accuracy was defined as the absolute difference between the programmed and SGRT‐measured amplitudes.

Because the SGRT system and the motion platform operate on independent, unsynchronized internal clocks, we used an approach similar to that of Roland et al.,^20^ in which the latency is recovered from the phase relationship between the reference and measured motion traces, without requiring a common time reference. The platform's start‐time timestamp from system logs was used to isolate the relevant motion segment from the continuous SGRT data stream, and the isolated SGRT data were fitted to a sinusoidal model, *y(t) = Asin((2πt/T) + ɸ)*, using a non‐linear least‐squares algorithm, where *y(t)* is the SGRT‐measured surface displacement at time *t(s), A*, is the motion amplitude (mm), *T*, is the period (s) of the programmed sinusoidal motion, and *ɸ*, is the phase shift (rad) between the SGRT‐measured and programmed waveforms. The phase shift *ɸ* extracted from the best‐fit parameters was used to calculate the SGRT system latency via Δt = −(*ɸ* / 2π) T. The resulting latency was evaluated against the AAPM TG‐302 recommended tolerance of 100 ms.^17^

### 2.4 SGRT + IGRT + triggered imaging (SITI) workflow

The SITI workflow addresses inter‐breath‐hold variability and intra‐fraction motion — two known limitations of DIBH — in real time. Patient positioning begins with SGRT‐based surface alignment, followed by volumetric IGRT verification in BH: an initial CBCT registration to bony anatomy is refined to the soft‐tissue target (if visible) or to a high‐density internal marker, and fluoroscopy is then used to confirm that the marker remains within its planned contour throughout the BH.

For intra‐fraction monitoring, Varian's triggered kV imaging operates concurrently with continuous SGRT surface tracking. A projection image is acquired before beam‐on and at 15° gantry intervals throughout each VMAT arc, providing multi‐angle coverage. On the DIBH CT, a high‐density internal marker clearly visible on projection images (e.g., implanted fiducial, surgical clip, calcification, or the visible tumor itself) is contoured and given an isotropic expansion of *x* mm to create the verification volume (e.g., “BH_Marker + *x*mm”), where x is based on the desired detection sensitivity for the residual displacements. The projection of this volume is overlaid on each live triggered image. If the therapist observes the marker projecting outside the volume on three consecutive acquisitions, the beam is manually held.

**FIGURE 2:**
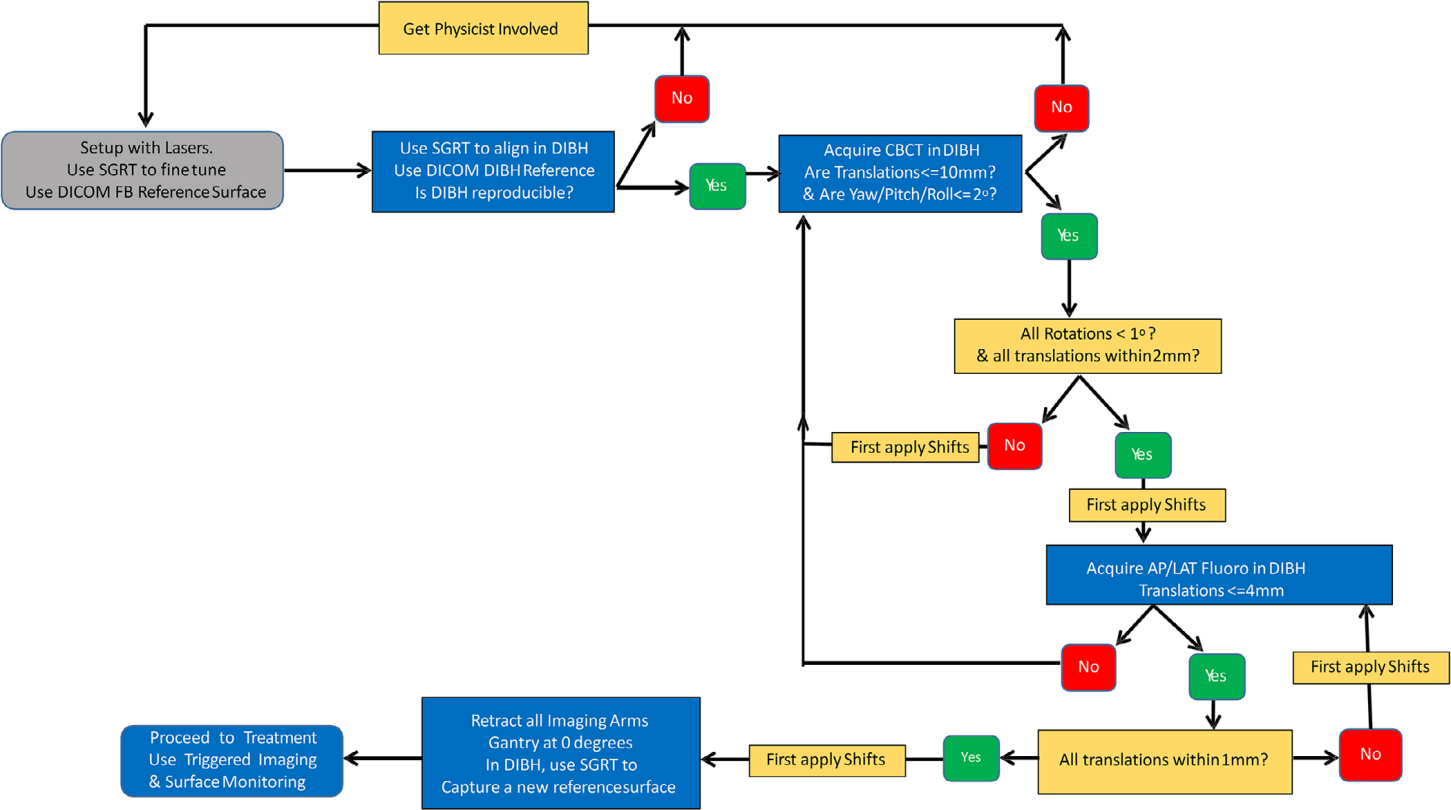
SGRT + IGRT + Trigger Imaging (SITI) Workflow of patient set‐up and localization until treatment delivery.

When a beam hold is initiated, the patient is re‐verified using CBCT—consistent with the initial volumetric IGRT verification step described above—to reassess both translational and rotational alignment before treatment resumes. Patients must sustain a BH for ≥20 s; longer BHs are preferred to permit single‐hold BH CBCT acquisition and larger uninterrupted beam segments. Triggered images should be acquired immediately upon beam resumption following any interruption. With triggered imaging configured on a gantry‐angle basis, the TrueBeam (v2.7) offers two interruption modes—‘beam‐off’ and ‘beam‐pause’—but only ‘beam‐off’ produces a trigger image immediately upon resumption. Ideal candidates are patients with reproducible 20–30 s BHs and a high‐density internal marker—verified as a reliable surrogate for tumor position—located in or near the tumor. The complete SITI workflow is shown in Figure 2.

### 2.5 End‐to‐end validation

**FIGURE 3:**
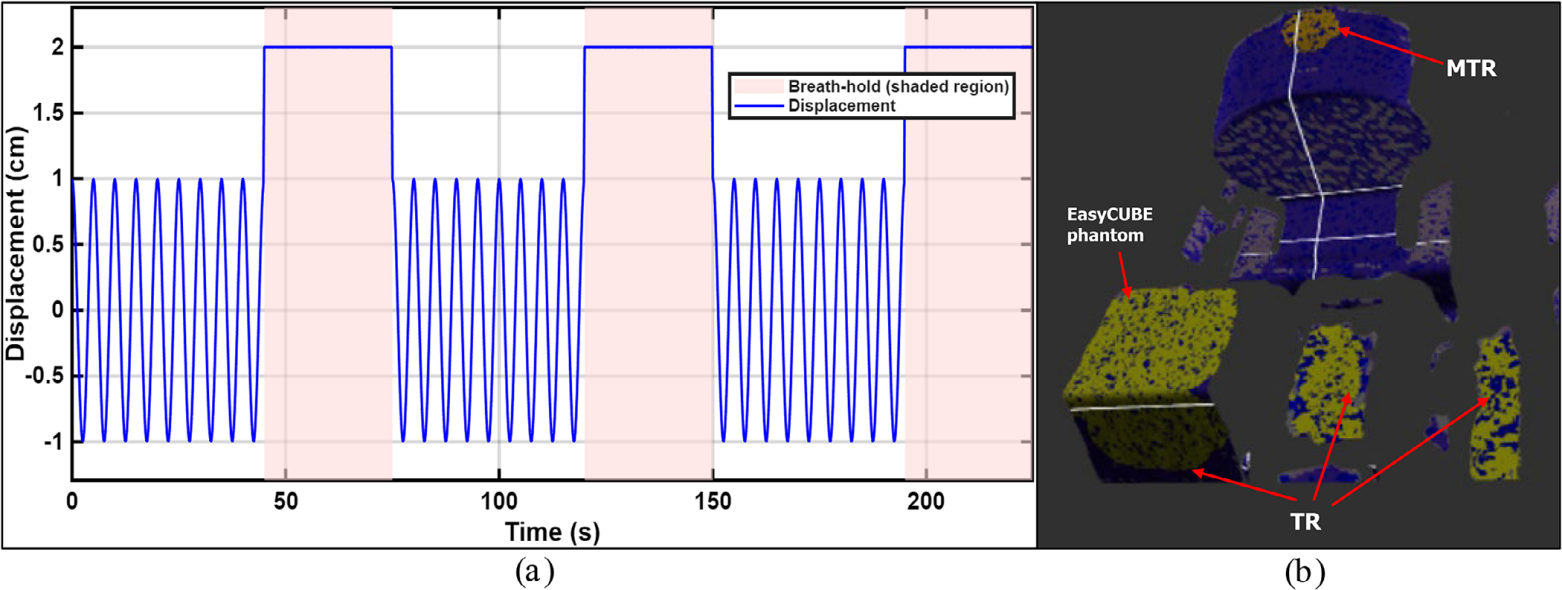
(a) Programmed motion waveform for the E2E test: a sinusoidal segment (5 s period) simulating free breathing, followed by a 30 s static segment simulating the breath‐hold plateau. (b) ROIs defined during CT simulation: the Motion Tracking ROI (MTR) on the CIRS E2E phantom (over an added sticky‐note surface feature) and the Treatment ROI (TR) on the adjacent EasyCUBE phantom and non‐moving couch portion.

To assess the operational feasibility and dosimetric impact of the SITI workflow, an end‐to‐end test was performed simulating a DIBH thoracic SBRT treatment. The CIRS E2E SBRT Phantom (Sun Nuclear, Melbourne, FL) was placed on the dynamic motion platform (Figure 1b). The platform provides one‐dimensional motion in the longitudinal direction; to introduce simultaneous vertical (anterior–posterior) motion, the phantom was mounted on an inclined foam wedge, producing a two‐dimensional motion trajectory. The programmed waveform consisted of a sinusoidal segment with a 5 s period (simulating free breathing) followed by a 30 s constant‐level static segment (simulating breath‐hold), as illustrated in Figure 3a. An Accredited Dosimetry Calibration Laboratory (ADCL)‐calibrated Exradin A16 (Standard Imaging Inc., Middletown, WI) small‐volume ionization chamber was inserted into the target volume of the CIRS phantom to measure point dose.

Prior to CT simulation, a live reference surface of the static phantom was captured using SGRT, and two ROIs — leveraging a specific capability of the LUNA 3D system to track multiple ROIs concurrently with distinct functional roles — were defined:

1. **Motion Tracking ROI (MTR)**: A small circular ROI (Figure 3b) used at CT simulation to confirm when the phantom was stationary in BH. Because the CIRS phantom lacks distinct 3D surface features in the thoracic region, a stack of sticky notes was taped to the surface as a trackable feature, around which the MTR was delineated. The MTR signal is not interfaced with the CT for automated gating.
2. **Treatment ROI (TR)**: A larger ROI defined on the adjacent EasyCUBE phantom (*placed on the static portion of the motion platform for the CT simulation only,)* and extending onto the portion of the treatment couch not subject to platform motion (Figure 3b). The TR served as a stable vertical baseline during CT simulation and as the global reference for set‐up and continuous positional verification during treatment. The EasyCUBE was removed prior to treatment delivery and is therefore not shown in Figure 1b.

The FB CT was acquired while the platform was executing the sinusoidal segment; the BH CT was initiated only after the SGRT system confirmed, via the MTR, that the phantom was within a ± 2 mm DIBH BH level tolerance window in the static segment. Both datasets were imported into the Eclipse™ TPS, where targets and OARs were contoured. The FB body contour was copied from the FB dataset onto the BH dataset to enable surface‐based initial setup in the treatment room.

Because the phantom did not contain implanted fiducials, the visible tumor insert was contoured on the BH dataset (lung window) and used directly as both the internal surrogate and the trigger‐image verification volume, with x = 0 mm (i.e., no isotropic expansion). This choice was deliberate: the tumor insert is sufficiently large that any non‐zero expansion would have rendered the verification volume insensitive to the small (1 and 2 mm) induced shifts evaluated in scenarios 3 and 4. Additionally, using the tumor contour directly preserved sensitivity to these displacements, which fall below the ± 3 mm SGRT tolerance and would not be flagged by surface monitoring alone. A VMAT SBRT plan (50 Gy in 5 fractions) was created and optimized on the BH dataset, then exported with its RT structure set to the LUNA 3D server.

In the treatment room, the phantom was first positioned to the FB DICOM reference surface using SGRT, then switched to the BH DICOM reference surface and guided into the BH level within the ± 2 mm tolerance window. The treatment was delivered four times, with the ion chamber measuring point dose for each scenario:

1. **SGRT‐only delivery**: Beam delivered without IGRT, gated only by the SGRT surface signal within the ± 2 mm BH level window.
2. **SITI workflow delivery**: The full SGRT + IGRT + triggered‐imaging workflow.
3. **SITI with 1 mm uncorrected shift**: An uncorrected 1 mm translational shift applied post‐IGRT in all three directions.
4. **SITI with 2 mm uncorrected shift**: An uncorrected 2 mm translational shift applied post‐IGRT in all three directions.

Shifts in scenarios 3 and 4 were applied as absolute couch translations in all three directions, independent of SGRT‐reported position, and intentionally left uncorrected to demonstrate the triggered‐imaging component's detection capability and to quantify the dosimetric impact. In a clinical scenario, any detected shift would be corrected by CBCT per the institutional workflow described above. For each of the four deliveries, the ion chamber reading was recorded and converted to a relative dose, with the SITI delivery serving as the in‐phantom reference (0.0%). The remaining three deliveries were expressed as percentage deviations in measured point dose relative to this reference. This relative‐dosimetry approach isolates the dosimetric impact of each workflow scenario from absolute‐dose calibration uncertainties associated with small‐volume chamber dosimetry in a non‐reference, heterogeneous SBRT phantom geometry.

## 3 RESULTS

### 3.1 Static localization accuracy

**FIGURE 4:**
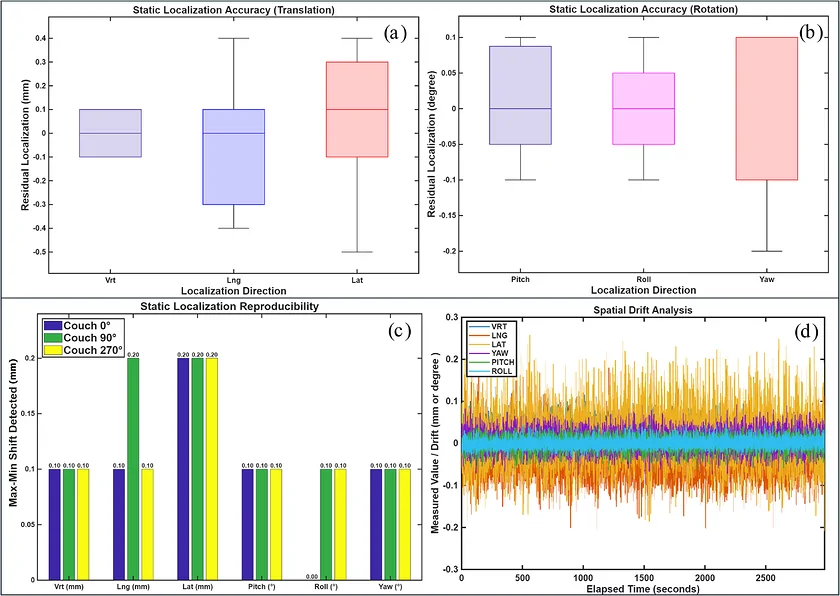
(a, b) Boxplots of static residual localization error for translation and rotation. (c) Static localization reproducibility (max–min shift). (d) Spatial drift of surface monitoring post system warm‐up.

The box plots of the residual localization error for the static localization accuracy tests are presented in Figure 4a,b. Overall translational displacement applied to the phantom covers a range ± 10 cm which is the AAPM TG‐302^17^ recommendation. The rotational displacement covers a range of ± 15°. This is because the maximum rotation that can be detected by the LAP LUNA 3D system is ± 15°. The maximum localization error was less than 0.5 mm for translation and less than 0.3° for rotation. These values are within the AAPM TG‐302^17^ tolerance for static localization accuracy of ≤ 1 mm for SRS/SBRT treatment using SGRT. However, from the plot, one can observe that many of the residual shifts were approximately zero.

### 3.2 Static localization reproducibility

The static localization reproducibility was quantified using the difference between the maximum and minimum SGRT obtained shifts for known couch shifts. This result is presented in Figure 4c. The reproducibility was within 0.2 mm for all translational directions and within 0.1 degrees for all rotational directions. The effect of couch angle on the reproducibility was also tested, and the result suggests that the couch angle does not affect the reproducibility of the system.

### 3.3 Post warmup spatial drift

Spatial drift is plotted in Figure 4d. Maximum translational and rotational drifts were +0.26 mm (lateral) and −0.1° (yaw). System stability, calculated from max–min drift, was 0.47 mm (translation) and 0.19° (rotation). Reproducibility during continuous monitoring, derived from maximum standard deviations, was 0.04 mm and 0.02°. The test was conducted only after camera temperature stabilization. Cameras are kept continuously powered (cycled only once annually) to avoid clinical use during warmup; no tracking instability was observed. These values establish a best‐case baseline for spatial drift during treatment.

### 3.4 LUNA 3D dynamic spatial localization and latency

**FIGURE 5:**
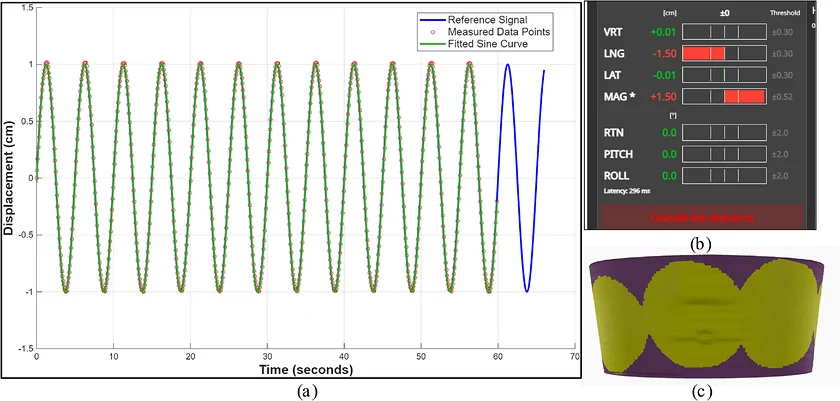
(a) Programmed (Reference Signal) and SGRT‐measured (Measured Data Points) motion traces with non‐linear least‐squares sinusoidal fit used to extract the phase shift and latency. (b) SGRT Real‐Time Deltas (RTDs) confirming motion restricted to the longitudinal axis. (c) Treatment ROI (TR) on the CIRS E2E phantom, used as the positional reference for SGRT‐based setup and monitoring.

Figure 5a shows the agreement between the programmed signal on the enhanced dynamic platform (Reference Signal) and the signal detected by the SGRT system (Measured Data Points). The peak‐to‐peak displacement in both cases was 2 cm, and the crest‐to‐crest time difference was 5 s, equal to the period of the programmed sinusoidal motion. The RTD display (Figure 5b) further confirmed that motion was restricted to the longitudinal direction, with no detected displacement in the vertical, lateral, or rotational axes.

The non‐linear least‐squares fit of the measured SGRT data yielded a phase shift of *ɸ* = −0.0397 rad, corresponding to a latency of Δt = 31.6 ms. The positive sign of Δt indicates that the SGRT‐reported trace lagged the reference signal, consistent with the expected acquisition and processing delay inherent to optical surface‐imaging systems. The measured latency is well below the 100 ms tolerance recommended by AAPM TG‐302, confirming that the system's temporal response is adequate for real‐time intra‐fraction motion monitoring and breath‐hold‐gated delivery.

### 3.5 End‐to‐end test

The results of the E2E validation are presented in Table 1, expressed as percentage deviations in measured point dose relative to the SITI delivery, which served as the in‐phantom reference (0.0%). Details of the VMAT plan used in the test — including arc geometry, energy, dose calculation algorithm, plan quality indices, and the local dose gradient at the chamber measurement point — are provided in Table S1.

**TABLE 1:** E2E dosimetric results expressed as percentage deviations in measured point dose relative to the SITI reference delivery.

Delivery scenario	Shift (mm)	Dose deviation vs. SITI (%)
SGRT‐only	0	0.0
SITI (reference)	0	0.0
SITI + 1 mm	1	−0.9
SITI + 2 mm	2	−2.2

Abbreviations: SGRT, Surface Guided Radiotherapy; SITI, SGRT + IGRT + Triggered Imaging.

**FIGURE 6:**
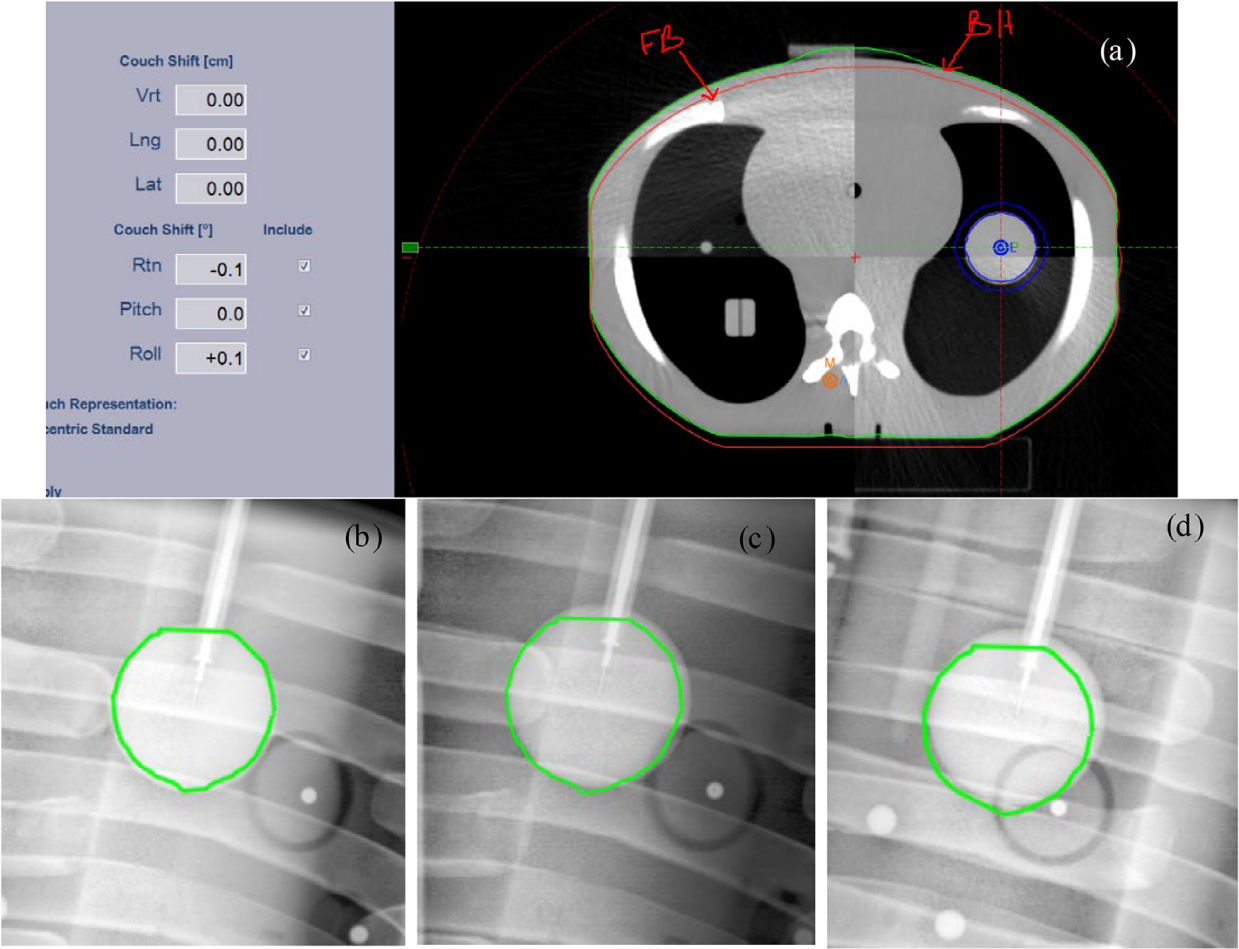
(a) Post‐SGRT CBCT verification of the BH setup prior to SITI delivery. Green and red contours = planning BH external and copied FB external; the offset reflects the A–P displacement coupled to the platform's S–I motion via the inclined wedge. Registration yielded 0.00 cm translations and rotations ≤0.1°, confirming negligible residual setup error. (b–d) Triggered kV images for (b) SITI delivery, (c) SITI + 1 mm induced shift, and (d) SITI + 2 mm induced shift, with the verification volume (green; tumor contour, x = 0 mm) overlaid on each live image. Panels (c) and (d) show the tumor deviating from the verification volume due to the induced shifts.

The SGRT‐only delivery agreed with the SITI reference to within 0.0%, confirming that under the static, rigid‐phantom conditions of the test, SGRT‐based BH gating alone produced a delivery geometrically equivalent to the full SITI workflow. This agreement was expected, as the use of a rigid phantom minimizes any discrepancy between the external surface and the internal target position, and was further supported by post‐SGRT CBCT verification showing negligible residual setup error (Figure 6a).

The clinical value of the SITI workflow was demonstrated in the scenarios with induced geometric errors. The 1 mm uncorrected 3D shift resulted in a measured point dose 0.9% lower than the SITI reference, and the 2 mm shift resulted in a 2.2% reduction — corresponding to ∼1%/mm, in close agreement with the local dose gradient at the chamber point derived from the TPS (Table S1), providing internal consistency between the measured impact and the planned dose distribution. These deviations therefore reflect the dose‐gradient characteristics of the specific VMAT plan used in this study rather than a universal dose–displacement relationship; for plans with more uniform target dose distributions, an equivalent shift may produce a smaller change in point dose, whereas plans with steeper dose gradients (e.g., SRS plans with high hotspots) may exhibit substantially larger deviations for the same geometric miss.

The induced shifts were clearly visualized on the triggered kV images, with representative examples acquired at selected gantry angles shown in Figure 6b–d. Because trigger images are acquired at 15° gantry intervals throughout the arc, any residual displacement that causes the visible tumor to project outside the verification volume on three consecutive acquisitions will be detected—independent of motion direction—enabling the therapist to manually hold the beam and initiate corrective action before treatment resumes.

## 4 DISCUSSION

This study commissioned the LAP LUNA 3D SGRT system and validated an integrated SITI) workflow for DIBH thoracic and abdominal SBRT on a Varian TrueBeam, in accordance with AAPM TG‐302 recommendations.

Static localization commissioning showed translational errors < 0.5 mm, rotational errors < 0.3°, and reproducibility within 0.2 mm/0.1° — meeting or exceeding AAPM TG‐302 tolerances for SGRT use in SRS/SBRT^17^. Post‐warmup spatial drift was minimal (< 0.47 mm; < 0.2°), supporting the practice of continuous system power to minimize thermal cycling. Dynamic testing confirmed sub‐millimeter spatial agreement and a latency of 31.6 ms, well below the AAPM TG‐302 100 ms tolerance^17^, ^19^—adequate for breath‐hold monitoring and real‐time surface guidance.

The E2E test established workflow feasibility and dosimetric response. Under static rigid‐phantom conditions, the SGRT‐only delivery agreed with the SITI reference to within 0.0%. The induced 1 and 2 mm 3D shifts produced point‐dose reductions of 0.9% and 2.2%, in close agreement with the ∼1%/mm local dose gradient at the chamber point derived from the TPS (Table S1). These values reflect the dose‐gradient characteristics of the specific VMAT plan tested and should not be generalized to plans with different gradient profiles; plans with more uniform target dose distributions may exhibit smaller dose changes for the same geometric shift, whereas plans with steeper dose gradients (e.g., SRS) may exhibit substantially larger deviations.

The 1 and 2 mm shifts lie below the ± 3 mm SGRT tolerance window and would not be flagged by surface monitoring alone. The triggered‐imaging component successfully visualized these sub‐tolerance displacements, enabling the therapist to manually hold the beam and initiate correction. This complementary role—surface guidance for initial setup and gating, triggered imaging for sub‐tolerance intra‐fraction verification—is the central operational rationale for the SITI workflow.  The two modalities are non‐redundant: SGRT provides the continuous external surrogate signal for breath‐hold gating, while triggered imaging provides periodic internal verification of sub‐tolerance displacements undetectable from the surface, triggering CBCT‐based correction.

Two important caveats apply to the end‐to‐end results. First, the phantom is a rigid, non‐deformable surrogate that does not capture patient‐specific factors such as deformable anatomy or variability in breath‐hold reproducibility. Second, in clinical practice the gross tumor is generally not sufficiently visible on planar kV images for reliable trigger‐image verification in thoracic or abdominal sites; implanted fiducial markers are typically required. The use of the visible tumor as the internal surrogate in this phantom study reflects only the absence of implanted markers in the CIRS E2E SBRT phantom and should not be interpreted as a clinically representative imaging condition.

The SITI workflow introduces additional imaging dose from three sources: The initial pre‐treatment CBCT, triggered kV projections acquired at 15° gantry intervals throughout each VMAT arc, and any repeat CBCT following a beam hold. A new CBCT is acquired once per beam‐hold event, and the frequency of such events depends on breath‐hold reproducibility. Patients with reproducible breath‐holds may complete a treatment session without additional CBCTs, while patients with less reproducible breath‐holds may require multiple re‐verifications per fraction. Each CBCT contributes an entrance dose on the order of mGy, while each triggered kV projection contributes on the order of tens of µGy; the cumulative additional dose per fraction therefore remains small relative to the SBRT therapeutic dose. Nevertheless, imaging dose should be included in the overall clinical risk–benefit assessment, particularly in patients requiring frequent beam holds. A comprehensive imaging‐dose evaluation was beyond the scope of this Technical Note and is identified as future work.

### 4.1 Scope and future clinical evaluation

This study was intentionally limited to technical commissioning and workflow validation; it does not quantify clinical outcomes, treatment efficiency, or patient compliance. These questions are best addressed through a prospective, IRB‐approved clinical study, which will examine treatment time, frequency of intra‐fraction interventions, BH reproducibility, and the potential for PTV margin reduction enabled by improved internal verification.

We also note that the LUNA 3D system is not currently interfaced with the Varian TrueBeam for automatic beam hold; the vendor is actively developing such an interface, which would enable fully automated beam‐hold functionality and further streamline the workflow in future implementations.

## 5 CONCLUSION

This study reports the commissioning of the LAP LUNA 3D SGRT system and the end‐to‐end validation of an integrated SGRT + IGRT + triggered‐imaging workflow on the Varian TrueBeam for DIBH thoracic and abdominal SBRT. Phantom results confirm sub‐millimeter localization accuracy, low spatial drift, sub‐100 ms latency, and dosimetric response consistent with the local plan dose gradient. Complementing prior reports on surface‐guided DIBH with triggered radiographic verification^15^ and dedicated commercial combined SGRT + IGRT platforms,^16^ this work provides a practical reference for implementing combined surface and internal‐anatomy verification on existing Varian TrueBeam infrastructure without dedicated proprietary hardware. Future IRB‐approved clinical evaluation will assess real‐world performance in thoracic and abdominal stereotactic treatments.

## AUTHOR CONTRIBUTION

**Alois Ndlovu**: Contributed to data collection; provided project oversight and supervision; and critically reviewed and edited the manuscript. **Offormata E. Osunkwor**: Co‐designed the study; acquired; analyzed; and interpreted the data. Drafted the original manuscript and prepared it for publication. **Roland Teboh**: Conceived and designed the study; contributed to data collection; analysis; and interpretation. He also provided critical feedback and participated in the revision and editing of the manuscript.

## CONFLICT OF INTEREST STATEMENT

The authors declare no conflicts of interest.

## ETHICAL APPROVAL

This study reports on the technical commissioning of a radiotherapy system using commercially available phantoms. No human participants or animals were involved in this research. Therefore, institutional review board (IRB) approval was not required for this work.

## Supporting information




Supporting Information

## Data Availability

The data that support the findings of this study are available from the corresponding author upon reasonable request.
