# Supplementary material for: Commissioning and end‐to‐end validation of a combined surface‐guided and triggered kV imaging workflow for breath‐hold SBRT on a Varian TrueBeam
Source: J Appl Clin Med Phys. 2026 Sep 18;27(10):e70812. doi: 10.1002/acm2.70812 (PMC13588323; doi:10.1002/acm2.70812)
Supplement: Supplementary file 1 [file ACM2-27-e70812-s001.docx]

**Table S1:** VMAT plan Details for the end-to-end phantom test.

| **Parameter** | **Value** |
| --- | --- |
| Prescription | 50 Gy in 5 fractions to the PTV |
| PTV volume | 59.2 cc |
| Number of arcs | 2 |
| Arc start/stop angles | 0.0/179 & 179/0.0 |
| Collimator angles | 30/330 |
| Beam energy | 6 MV FFF |
| Maximum dose rate | 1400 MU/min |
| Dose calculation algorithm | AAA |
| Calculation grid size | 1.25 mm |
| Heterogeneity correction | Enabled |
| Conformity Index (CI) | 0.966 |
| Homogeneity Index (HI) | 1.48 |
| Gradient Index (GI) | 3.592 |
| Chamber location relative to PTV | geometric center of PTV |
| Local dose gradient at chamber point | 1 %/mm |
